# Temporal trends and correlates of overall and domain-specific sitting time in Germany between 2014 and 2023

**DOI:** 10.1186/s12889-025-26005-9

**Published:** 2025-12-30

**Authors:** Birgit Wallmann-Sperlich, Claas Lendt, Bianca Biallas, Hansjörg Baurecht, Carmen Jochem, Jens Bucksch, Michael Leitzmann, Ingo Froboese

**Affiliations:** 1 Integrative and Experimental Exercise Science and Training, Institute for Sports Science, Faculty of Human Science, Julius-Maximilians University of Würzburg, Judenbühlweg 11, Würzburg, D-97082 Germany; 2 Department of Epidemiology and Preventive Medicine, University of Regensburg, Franz-Josef-Strauss-Allee 11, Regensburg, D-93053 Germany; 3 Institute of Movement Therapy and Movement-oriented Prevention and Rehabilitation, German Sport University Cologne, Am Sportpark Müngersdorf 6, Cologne, 50933 Germany; 4 Medical Sociology, Department of Epidemiology and Preventive Medicine, University of Regensburg, Franz-Josef-Strauss-Allee 11, Regensburg, 93053 Germany; 5 Chair of Planetary & Public Health, University of Bayreuth, Universitätsstraße 30, Bayreuth, D-95447 Germany; 6 Department of Prevention and Health Promotion, Faculty of Natural and Sociological Sciences, Heidelberg University of Education, Postfach 10 42 40, Heidelberg, D-69032 Germany

**Keywords:** Sedentary behavior, Sitting time, Temporal trends, Domain-specific sitting, Correlates of sedentary behavior, Physical activity, Population surveillance, Population-based study, Germany, Cross-sectional survey

## Abstract

**Background:**

Sedentary behavior, particularly prolonged sitting, is a significant global public health issue. However, comprehensive population-level temporal trends on sitting time are sparse, hampering effective monitoring and policy development. This study aims to examine temporal trends and correlates of overall and domain-specific sitting time among the adult population in Germany from 2014 to 2023.

**Methods:**

Data were obtained from five cross-sectional, population-representative telephone surveys conducted in Germany in 2014, 2016, 2018, 2021, and 2023, comprising a total of *N* = 14,417 adults aged 18 years and older. Participants completed the domain-specific Marshall Sitting Questionnaire, reporting sitting time across five domains: transport, work, television viewing, leisure-time electronic media use, and other leisure activities. Descriptive statistics and linear regression analyses were performed to examine time trends and sociodemographic and lifestyle correlates, with all results presented as mean estimates and 95% confidence intervals.

**Results:**

Between 2014 and 2023, average overall weekday sitting time increased by 76 minutes per weekday, rising from 457 to 533 minutes. The most pronounced relative increases were observed in work-related (42%) and leisure-time electronic media use (31%). The prevalence of high sitting time (>8 hours/weekday) rose from 42% in 2014 to 55% in 2023. Regression analyses indicated that men (31.8 minutes/weekday, 95% CI: 22.5, 41.1), younger adults (-2.3, 95% CI: -2.5, -2.0), individuals with higher education (57.1, 95% CI: 43.8, 70.4) and income levels (41.7, 95% CI: 25.9, 57.5), and urban residents (21.5, 95% CI: 8.7, 34.2) had significantly longer overall sitting times compared to women, older adults, individuals with lower education and income, and those living in rural areas, respectively.

**Conclusions:**

Temporal trends from Germany indicate a substantial increase in both overall and domain-specific sitting time between 2014 and 2023, predominantly driven by work-related and screen-based leisure activities. These findings underscore the urgent need for public health strategies aimed at reducing prolonged sitting, particularly in work and leisure contexts. Targeted population-specific and context-sensitive interventions are increasingly needed to mitigate the rise in overall and domain-specific sitting time.

**Supplementary Information:**

The online version contains supplementary material available at 10.1186/s12889-025-26005-9.

## Background

Sedentary behavior is a significant and independent health risk, strongly linked to increased morbidity and mortality [1–3]. It is defined as any waking behavior in a seated or reclining body position with an energy expenditure of less than 1.5 metabolic equivalents of task (METs) [4]. Prolonged sitting – typically defined as sitting more than eight hours per day - has been shown to increase the risk of all-cause mortality [5]. Evidence further indicates that this risk can be partially outweighed by higher levels of moderate-to-vigorous physical activity (MVPA) and may be eliminated entirely with high levels of self-reported MVPA, specifically 60 to 75 min per day [5].

However, a large portion of the German population does not reach recommended levels of MVPA [6], with only 43% of women and 48% of men meeting the minimal aerobic physical activity guidelines [7]. This leaves most of the population - those who are inactive or engage only in the minimum recommended amount - at an increased risk of illness and premature mortality associated with prolonged sitting.

Temporal trend data on sedentary behavior can help assess and monitor population sitting time across different domains (work, transport, TV viewing, other electronic media use, leisure), identify high-risk groups, and inform and guide global and national public health strategies. Comparable trend data are especially valuable for tracking changes over time. Studies from Europe and the United States show differing trends. Eurobarometer data indicated a decline in sitting time in Europe until 2013, followed by stabilization thereafter, although this trend should be interpreted with caution due to a change in survey methodology in 2013 [8, 9]. In contrast, analyses of the National Health and Nutrition Examination Survey (NHANES) data in the United States showed an increase in adults’ overall sitting time from 2001 to 2016, largely driven by greater computer use [10]. A subsequent study covering 2007/2008 − 2017/2018 confirmed a continued overall increase, although a decline was observed after 2013/2014 [11]. These inconsistencies underscore the need for country-specific and domain-specific analyses, particularly in light of recent changes in daily life, such as increased digitalization of work and leisure, changing leisure-time preferences, as well as the impact of the COVID-19 pandemic [12–14].

Furthermore, identifying sociodemographic and behavioral correlates of both overall and domain-specific sitting time is essential to understand which population subgroups, and in which contexts, are most at risk of prolonged sitting time. Previous research has shown that sitting time - both in overall and within specific domains - varied by age, gender, education, employment status, and physical activity levels [15–17]. Exploring these correlates offers valuable insights into the social distribution of sedentary behavior and can support the development of more targeted public health interventions aimed at reducing sitting time in specific subgroups and contexts.

Using nationally representative data from Germany across five cross-sectional waves between 2014 and 2023, all employing the same assessment and methodological approach, this study had two main objectives: (i) to investigate temporal changes in overall and domain-specific sitting times; and (ii) to identify socio-demographic and behavioral factors associated with these trends.

## Methods

### Study design

This study is based on data from five independent cross-sectional surveys of the German Health Insurance Company DKV Health Report [18], a nationally representative survey conducted biennially in Germany since 2010. Data were collected using computer-assisted telephone interviews (CATI) administered by trained professional market research center interviewers. The surveys were typically conducted between February and April in each wave, thereby minimizing potential seasonal bias. Each interview lasted between 20 and 25 min, and covered various aspects of health-related behavior, including physical activity, sitting time (assessed since 2014), demographics, self-rated health status, and other health-related topics. Pre-tests were conducted before each survey wave, during which selected professional interviewers were trained to administer the computer-assisted standardized questionnaire. Each survey was approved by the Ethics Committee of the German Sport University Cologne.

### Study population

For this analysis, five survey waves conducted between 2014 and 2023 were considered, comprising a total of 14,417 participants, with an average of approximately 2,883 respondents per wave. Participants were drawn from all 16 German federal states and were 18 years or older.

The sample was drawn from the ADM Pool for Telephone Samples (ADM = Arbeitskreis Deutscher Markt- und Sozialforschungsinstitute) — a consortium of German market and social research institutes. The ADM pool represents a precisely coordinated national sampling frame that includes all potential telephone numbers in Germany and serves as the basis for selecting representative population samples. The sample was stratified by age and gender and replicated 1:1. Post-stratification weights were applied for each survey year using reference data from the German Federal Statistical Office. These weights accounted for gender, age group, municipality size, and educational attainment to obtain a representative statement. The response rates varied across waves, ranging from 13.0% in 2014, 13.5% in 2016, 11.0% in 2018, 7.0% in 2021, to 4.6% in 2023.

### Measures

#### Sitting time

To assess sitting time, we used the Marshall Sitting Questionnaire [19], translated into German, and focused on weekdays. The questionnaire includes five items measuring time spent (in hours and minutes) on specific sitting activities across the following domains: (a) transportation (e.g., commuting to work or shopping); (b) work-related sitting; (c) watching television; (d) computer and other electronic media use at home; and (e) leisure activities that do not include television or electronic media use (e.g., social visits, going to the movies, dining out). The questionnaire shows good reliability for weekday sitting time in the work, television, and electronic media use domains (*r* = 0.78–0.84) and lower reliability in the transport and other leisure domains (*r* = 0.34–0.60). Validity, assessed against log data and sedentary accelerometer data, was highest for weekday sitting time at work (*r* = 0.69) and electronic media use at home (*r* = 0.74) and lowest for other leisure (*r* = 0.21–0.26) [19].

Cases with an overall sitting time exceeding 16 h per weekday, as well as those with missing values across all items, were excluded from the analysis (2014 (*n* = 181, 6%), 2016 (*n* = 138, 5%), 2018 (*n* = 133, 5%), 2021 (*n* = 303; 11%) and 2023 (*n* = 17, < 1%) (Supplement Table S1). In cases where at least one sitting domain contained valid data, missing values for the remaining domains were set to zero, under the assumption that non-response indicated no sitting time in those specific domains.

### Sociodemographic, anthropometric, and physical activity variables

Variables included self-reported age, sex, weight, height, education, household income, community size, and physical activity. Body mass index (BMI) was calculated from self-reported body weight and height using the standard formula BMI = weight (kg)/height (m)^2^. Education was categorized into no formal qualification, lower secondary school graduation (9 years of education), secondary school diploma (10 years), and high school diploma (13 years or more). Income was grouped into low (< €2,000), middle (€2,000–<€4,000), and high (≥€4,000) monthly net household income. Community size was categorized as < 20,000, 20,000–500,000, and > 500,000 inhabitants. Physical activity was assessed using the Global Physical Activity Questionnaire (GPAQ), capturing activity in three domains over a typical week: work (both paid and unpaid), transport (e.g., walking and cycling for commuting), and leisure (e.g., sports, active recreation) [20]. Based on GPAQ scoring guidelines, physical activity was converted to MET-minutes per week and categorized according to WHO recommendations into insufficiently active (< 600 MET-min/week) and sufficiently active (≥ 600 MET-min/week) [21].

### Data processing and statistical analyses

Data processing and statistical analysis were conducted using R (version 4.4.2) [22]. For both the unweighted (supplement Table S2 and Table S3) and weighted total samples, sitting time (overall and by domain) was descriptively analyzed for each survey year using mean values and 95% confidence intervals (CI). The prevalence of high sitting time (> 8 h per weekday) was calculated, with this threshold chosen based on evidence associating it with an increased mortality risk [5].

Statistical analysis was performed using multiple linear regression. Sitting time in minutes per weekday (overall and domain-specific) served as the dependent variable. Independent variables included survey year, along with sociodemographic and behavioral characteristics (physical activity as MET-minutes). To examine temporal trends, we compared models including a simple linear main effect for survey year with models incorporating a natural cubic spline term. Model fit was assessed using AIC values and likelihood ratio tests to evaluate potential non-linearity. For sitting time during watching TV and during electronic media use, spline models showed a better fit than linear models (*p* < 0.05; Supplement Table S4), and these were therefore adopted. For these two domains, average marginal effects were computed to estimate the annual change in sitting time. Additionally, we calculated McFadden’s pseudo R² to assess the goodness of fit for each model (Supplement Table S5) and conducted analyses of collinearity (Supplement Table S6).

To address missing data in BMI, income, and physical activity minutes per day, multiple imputation was performed using the Multivariate Imputation by Chained Equations (MICE) algorithm, as implemented in the R *mice* package (version 3.17.0) [23]. Ten imputed datasets were generated with 5 iterations each. Regression analyses were conducted using the *svyglm* function from the R survey package (version4.4-2.4.4.4; Lumley et al., 2024), taking into account the complex survey design. Details on missing data by variable and survey year are provided in the Supplement as well as the results of sensitivity analyses of regression models using only complete cases (without imputation of missing values) (see Supplement Table S1 and Table S7).

## Results

### Sample characteristics

Sample characteristics across survey years are presented in Table 1. The sex distribution remained largely stable, with a slight increase in the proportion of women observed in 2023. The age distribution shifted modestly, with a growing proportion of adults aged 80 years and older (up to 99 years) and a decline in younger participants aged 18–29. The prevalence of normal BMI (18.5 to 24.9) increased, while overweight (BMI: 25.0 to 29.8) showed a slight decline. Smoking prevalence decreased over time, and adherence to physical activity guidelines remained relatively high and stable. Higher levels of educational attainment and household income increased across survey years.

**Table 1:** Distribution of participant characteristics across survey years 2014 to 2023

	Survey year				
Characteristic	2014 *N* = 3,102	2016 *N* = 2,830	2018 *N* = 2,885	2021 *N* = 2,800	2023 *N* = 2,800
Sex					
Female	1,581 (51%)	1,448 (51%)	1,476 (51%)	1,433 (51%)	1,481 (53%)
Male	1,521 (49%)	1,382 (49%)	1,409 (49%)	1,367 (49%)	1,319 (47%)
Age (years)					
18–29	503 (16%)	443 (16%)	462 (16%)	430 (15%)	376 (13%)
30–44	699 (23%)	636 (22%)	647 (22%)	654 (23%)	646 (23%)
45–64	1,143 (37%)	1,044 (37%)	1,047 (36%)	989 (35%)	998 (36%)
65–79	582 (19%)	531 (19%)	533 (18%)	510 (18%)	525 (19%)
≥80	175 (5.6%)	177 (6.3%)	196 (6.8%)	218 (7.8%)	255 (9.1%)
Body Mass Index (WHO categories)					
Underweight (< 18.5 kg/m²)	46 (1.5%)	125 (4.5%)	73 (2.6%)	87 (3.1%)	70 (2.5%)
Normal weight (18.5–24.9 kg/m²)	1,417 (47%)	1,250 (44%)	1,335 (47%)	1,337 (48%)	1,411 (51%)
Overweight (25.0–29.9 kg/m²)	1,052 (35%)	1,027 (36%)	943 (34%)	894 (32%)	873 (31%)
Obesity (≥ 30.0 kg/m²)	523 (17%)	411 (15%)	465 (16%)	470 (17%)	437 (16%)
Unknown	63	17	70	12	9
Current smoking	757 (24%)	649 (23%)	683 (24%)	758 (27%)	543 (19%)
Physical activity guideline adherence (≥ 600 MET*min)	2,620 (87%)	2,230 (80%)	2,311 (81%)	2,228 (80%)	2,330 (84%)
Unknown	81	58	17	1	29
Education					
No educational qualification	101 (3.2%)	109 (3.8%)	121 (4.2%)	119 (4.3%)	95 (3.4%)
Lower secondary school (9 yrs)	1,095 (35%)	920 (33%)	876 (30%)	854 (30%)	660 (24%)
Secondary school (10 yrs)	956 (31%)	872 (31%)	895 (31%)	867 (31%)	927 (33%)
High school or university (≥ 13 yrs)	950 (31%)	929 (33%)	992 (34%)	960 (34%)	1,117 (40%)
Household income (€ per month)					
<2,000	1,674 (62%)	477 (43%)	661 (44%)	681 (43%)	372 (26%)
2,000-<4,000	823 (31%)	478 (43%)	638 (42%)	617 (39%)	696 (49%)
≥4,000	197 (7.3%)	144 (13%)	206 (14%)	271 (17%)	363 (25%)
Unknown	407	1,731	1,381	1,231	1,369
Community size (no. of inhabitants)					
<20,000	1,193 (38%)	1,086 (38%)	1,091 (38%)	1,065 (38%)	1,088 (39%)
20,000–500,000	1,358 (44%)	1,222 (43%)	1,254 (43%)	1,211 (43%)	1,205 (43%)
>500,000	551 (18%)	522 (18%)	540 (19%)	524 (19%)	507 (18%)

Values are based on weighted samples and presented as absolute numbers (*n*) and percentages (%)

### Descriptives of overall and domain-specific sitting times

**Fig. 1:**
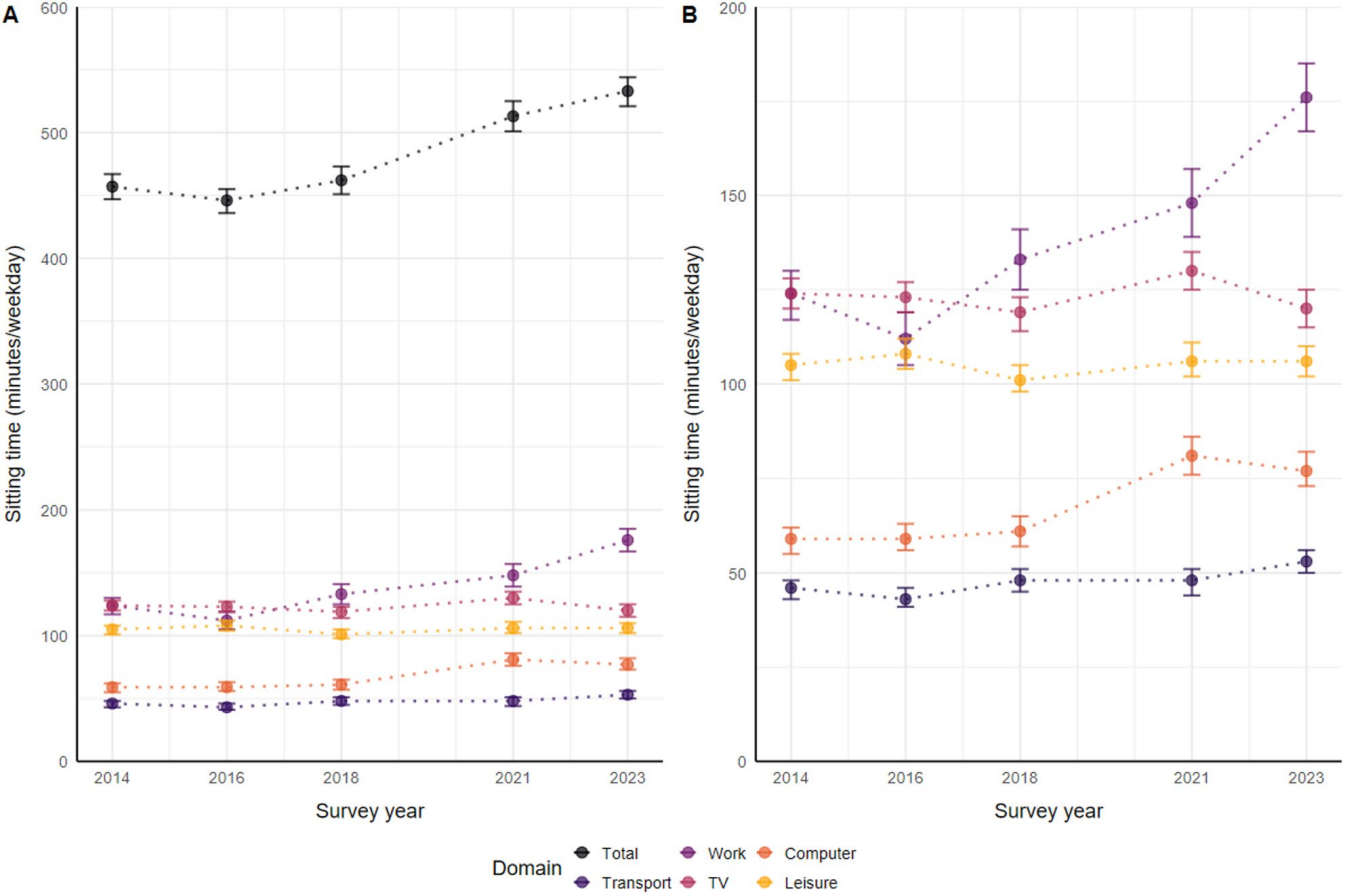
**A** Overall and domain-specific sitting time across survey years, and (**B**) a zoomed-in view of domain-specific sitting time

Overall sitting time increased on average by approximately 76 min per weekday (16.6%) between 2014 and 2023, rising from 457 min per weekday in 2014 (95% CI: 447–467) to 533 min per weekday in 2023 (95% CI: 521–544). This increase was primarily attributable to longer durations of sitting time for work (41.9%), leisure-time electronic media use (30.5%), and transport (15.2%), while television viewing time remained relatively stable. The steepest increase in average sitting time occurred between 2018 and 2021, rising from 462 min per weekday in 2018 (95% CI: 451–473) to 513 min per weekday in 2021 (95% CI: 501–525). The prevalence of high sitting times (> 8 h per weekday) increased from 42% (95% CI: 40%, 44%) in 2014 to 55% (95% CI: 53%, 58%) in 2023. Descriptive statistics are presented in Table 2; Fig. 1.

**Table 2:** Overall and domain-specific sitting time and prevalence of high sitting time (> 8 h/weekday) among adults in Germany across survey years 2014 to 2023 (mean minutes per weekday, 95% confidence intervals)

Domain	Survey year				
	2014 *N* = 3,102	2016 *N* = 2,830	2018 *N* = 2,885	2021 *N* = 2,800	2023 *N* = 2,800
Overall	457 (447, 467)	446 (436, 455)	462 (451, 473)	513 (501, 525)	533 (521, 544)
Transport	46 (43, 48)	43 (41, 46)	48 (45, 51)	48 (44, 51)	53 (50, 56)
Work	124 (117, 130)	112 (105, 119)	133 (125, 141)	148 (139, 157)	176 (167, 185)
Television	124 (120, 128)	123 (119, 127)	119 (114, 123)	130 (125, 135)	120 (115, 125)
Electronic media use at home	59 (55, 62)	59 (56, 63)	61 (57, 65)	81 (76, 86)	77 (73, 82)
Leisure time, other	105 (101, 108)	108 (104, 112)	101 (98, 105)	106 (102, 111)	106 (102, 110)
Prevalence > 8 h sitting (%)	42% (40%, 44%)	38% (36%, 40%)	42% (40%, 45%)	51% (48%, 53%)	55% (53%, 58%)

Values are based on weighted samples

### Temporal, socio-demographic, and behavioral correlates of overall and domain-specific sitting time

Over the study period, overall sitting time increased by an average of 8.7 min per weekday per calendar year (95% CI: 7.3, 10.1). This trend was primarily driven by longer sitting times during work and electronic media use at home, which rose by an average of 4.5 min (95% CI: 3.5, 5.6) and 1.5 min per weekday (95% CI: 1.0, 2.0), respectively.

Among sociodemographic factors, men sat on average 31.8 min longer per weekday than women (95% CI: 22.5, 41.1). However, women reported more time spent sitting during leisure activities, with an average of 5.8 additional minutes per weekday (95% CI: 2.1, 9.5) compared to men. Age was inversely associated with overall sitting time, decreasing by an average of 2.3 min per year of age (95% CI: −2.5, −2.0). This decline was most pronounced for work-related sitting time (−2.8, 95% CI: −3.0, −2.6), whereas television-related sitting time was higher among older adults (1.2 min per weekday, 95% CI: 1.0, 1.3). Higher BMI was associated with higher sitting time. Each one-unit increase in BMI corresponded on average to an additional 2.2 min of overall sitting per weekday (95% CI: .1.3, 3.1), mainly driven by higher television viewing time (2.1 min/weekday, 95% CI: 1.6, 2.5).

Education level was also associated with sitting time. Individuals with 13 or more years of schooling exhibited on average higher overall sitting time (57.1, 95% CI: 43.8, 70.4) and work-related sitting time (70.8, 95% CI: 61.4, 80.2), but lower television-related sitting time (−28.5, 95% CI: −34.1, −22.9), compared to those with lower secondary education (9 years of schooling). Similarly, individuals with a net monthly household income of €4,000 or more spent 41.7 min more overall sitting per weekday (95% CI: 25.9, 57.5) than those earning less than €2,000.

Urbanization was positively associated with sitting time. Individuals residing in cities with more than 500,000 inhabitants reported a higher average overall sitting time (21.5, 95% CI: 8.7, 34.2) compared to those living in smaller towns with fewer than 20,000 residents. This difference was particularly evident for sitting time during electronic media use (9.9, 95% CI: 5.3, 14.6) and other leisure-related sitting time (8.2, 95% CI: 3.4, 13.0).

An inverse relationship was observed between physical activity and sitting time. For every additional minute of moderate-to-vigorous physical activity per day, overall sitting time decreased by an average of 0.1 min (95% CI: −0.2, −0.1) or 8.4 s (Table 3).

**Table 3:** Association of temporal, sociodemographic, and behavioral correlates with overall and domain-specific sitting time. Results from multiple linear regression models (B = estimated difference in minutes per weekday; 95% confidence intervals) with significant results in bold print, *Calendar year was modelled using natural cubic splines showing the average marginal effect to estimate annual change in sitting time

	Domain					
Variable	Overall	Transport	Work	Television*	Electronic media use at home*	Leisure time, other
Calendar year (2014–2023)	**8.71 ** **(7.28**,** 10.14)**	**0.87 ** **(0.45**,** 1.3)**	**4.53** **(3.49**,** 5.57)**	−0.2 (−0.74, 0.34)	**1.49** **(1**,** 1.97)**	0.18 (−0.36, 0.72)
Male	**31.76** **(22.47**,** 41.05)**	**11.8** **(9.17**,** 14.44)**	**7.16** **(0.62**,** 13.7)**	1.68 (−2.16, 5.52)	**17.01 (13.48, 20.55)**	−5.8 (−9.52, −2.07)
Age (years)	**−2.25** **(−2.54**,** −1.96)**	**−0.13** **(−0.21**,** −0.05)**	**−2.81** **(−3.01**,** −2.6)**	**1.15** **(1.03**,** 1.28)**	**−0.9** **(−1.02**,** −0.78)**	**0.44** **(0.33**,** 0.55)**
Body mass index (kg/m^2^)	**2.17** **(1.25**,** 3.1)**	−0.15 (−0.41, 0.11)	−0.02 (−0.64, 0.61)	**2.08** **(1.64**,** 2.52)**	0.35 (−0.25, 0.95)	−0.1 (−0.48, 0.29)
Secondary school (10 years)	**18.17** **(5.03**,** 31.3)**	0.53 (−3.41, 4.47)	**26.08 (17.07, 35.09)**	**−12.89** **(−18.44**,** −7.34)**	**5.71 ** **(0.96**,** 10.46)**	−1.36 (−6.51, 3.79)
High school or university (≥ 13 years)	**57.09** **(43.77**,** 70.41)**	−1.67 (−5.62, 2.27)	**70.82 (61.43, 80.2)**	**−28.5** **(−34.09**,** −22.9)**	**19.66 (14.66, 24.66)**	−3.1 (−8.24, 2.04)
No formal educational qualification	**−54.12** **(−93.86**,** −14.38)**	**−16.16** **(−23.1**,** −9.23)**	−17.39 (−41.39, 6.62)	−16.12 (−34.51, 2.27)	7.45 (−10.62, 25.51)	−12 (−27.89, 3.89)
€2,000-<€4,000 monthly income	**14.36** **(2.6**,** 26.12)**	2.59 (−0.99, 6.18)	**38.38 (29.41, 47.34)**	**−11.09** **(−15.51**,** −6.66)**	**−9.14** **(−13.34**,** −4.93)**	**−6.09** **(−10.87**,** −1.3)**
≥ €4,000 monthly income	**41.74** **(25.94**,** 57.53)**	**4.66** **(0.28**,** 9.04)**	**81.65 (69.98, 93.32)**	**−21.01** **(−27.15**,** −14.87)**	**−10.66** **(−18.37**,** −2.96)**	**−12.69** **(−19.22** **,** ** −6.17)**
Mid-sized town (20,000–500,000,000 inhabitants)	7.4 (−2.76, 17.56)	**−3.24 (−6.09**,** −0.39)**	−0.22 (−7.46, 7.03)	2.3 (−1.91, 6.51)	**5.53** **(1.75**,** 9.31)**	3.28 (−0.73, 7.3)
Large city (> 500,000 inhabitants)	**21.47** **(8.71**,** 34.24)**	0.17 (−3.67, 4.02)	5.01 (−3.65, 13.67)	−1.84 (−6.88, 3.2)	**9.94** **(5.27**,** 14.61)**	**8.23** **(3.44**,** 13.03)**
MVPA (minutes per day)	**−0.14** **(−0.17**,** −0.11)**	0.02 (0.01, 0.03)	**−0.15** **(−0.17**,** −0.13)**	**−0.02 (−0.03, −0.01)**	−0.01 (−0.02, 0)	**0.01** **(0**,** 0.02)**

Calendar year, age, body mass index, and MVPA were modeled as continuous variables

Reference groups: women; lower secondary school (9 years); small town (<20,000 inhabitants); <€2,000 monthly income

*MVPA* moderate-to-vigorous physical activity

## Discussion

This study used repeated, independent cross-sectional surveys from a nationwide representative sample of adults in Germany (2014–2023) to examine long-term trends in sitting time, allowing us to identify relevant changes in both overall and domain-specific weekday sitting time. Overall sitting time increased by an average of 76 min per weekday, with the most pronounced rise occurring between 2018 and 2021. This increase was particularly marked among men, younger adults, individuals with higher education, and urban residents. The largest relative increases were observed in transport-related sitting (+ 15.2%), workplace sitting (+ 41.9%), and electronic media use at home (+ 30.5%). The rising prevalence of prolonged sitting reflects a notable shift in physical behavior patterns.

While our findings concerning trends in sitting time partially align with international studies reporting increases in sedentary time, particularly those conducted during or following the COVID-19 pandemic, they also reveal patterns that diverge from previous research. Studies from countries such as Mexico [24] and parts of Asia, such as Korea [25], have reported rising trends in sitting time. In contrast, data from North America [10, 11], several European countries [8, 9], Australia [26], and global pooled analyses [27] suggest more stable or even declining trends. Some of these studies report periods of stagnation [26] or initial increases followed by declines, as observed in U.S. NHANES data [11].

Over the nine-year observation period, our study identified a notably high and increasing prevalence of prolonged sitting (> 8 h per weekday), rising from 42% in 2014 to 55% in 2023. These figures markedly exceed the 19.5% reported in recent German surveillance data [28]. While this trend is consistent with international observations of increasing sedentary behavior [24, 29], our estimates are considerably higher than those reported in most international studies, yet remain somewhat lower than the prevalence observed in Korea [25]. This discrepancy may be partly explained by differences in measurement approaches. Our study assessed domain-specific self-reported sitting time across five distinct domains, whereas most studies relied on a single-item measure of overall sitting time [8, 9, 11, 24, 25] or other less detailed measures [10, 26]. A systematic review has shown that single-item self-report measures tend to underestimate sitting time [30], which may account for the lower prevalence observed in those studies. In contrast, the higher prevalence reported in Korea [25] may reflect different lifestyle or occupational patterns. Additionally, objective studies using accelerometry from European populations have shown that actual sitting times are substantially higher than self-reported estimates [31], further suggesting that sedentary time has been systematically underestimated in previous research. In addition, declining response rates in our telephone survey over time – an issue also documented in other European studies [32] - may also have contributed to the relatively high sitting times and the observed prevalence of sitting more than 8 h per weekday, introducing potential non-response bias. Although post-stratification weights were applied to adjust for sociodemographic differences, such weighting cannot fully account for the risk of selective response.

Regarding the long-term observation period, which includes the full COVID-19 pandemic timeline, we observed the steepest increase between 2018 and 2021, mainly driven by increased sitting during electronic media use at home, work, and television viewing, followed by a continued but more gradual rise in 2023. This pattern is consistent with emerging research documenting pandemic-related increases in sitting time [12–14], although most prior studies have focused on short-term changes or relied on single cross-sectional designs. Our findings extend this body of evidence by providing a broader view of population-level changes in sitting time over nearly a decade. It remains uncertain whether this upward trend will continue beyond 2023 in Germany and whether similar patterns will emerge in future international trend studies.

Work-related sitting constitutes the largest portion of daily sedentary time among adults [33, 34], a trend reflected in our findings, with an increase from 27.1% in 2014 to 33.0% in 2023. This is consistent with previous research reporting a median occupational sitting time of 4.2 h per day, with a range of 1.9 to 6.4 h per day [27]. While our data do not allow for a direct analysis of causal factors, the observed increase in occupational sitting may be linked to broader labor market trends, such as the rise in screen-based work and technological automation, as discussed in previous literature [35–38].

Leisure-related sitting times – including TV viewing, electronic media use at home, and other leisure activities - increased slightly from 288 min per weekday in 2014 to 303 min in 2023. However, the trends across the different domains diverged: while sitting time during other leisure activities remained stable, and time spent sitting while watching TV slightly decreased, a noticeable increase was observed in leisure-time use of computers or other electronic media. These sitting patterns point to a broader societal shift in screen-based behaviors: the growing use of social media, mobile phones, online streaming services, and computer games is gradually replacing traditional forms of media consumption, such as conventional television viewing [39]. This shift reflects a transformation in how leisure time is spent and highlights the growing role of digital technologies in everyday life.

Transport-related sitting times showed a slight increase in our study of the German population, mirroring similar trends observed in China, where passive transportation behaviors are on the rise [40]. Interestingly, this increase partly contrasts with findings on commuting behavior among Swedish workers, indicating a shift towards more active commuting modes [41]. While these trends may seem contradictory, especially in light of national initiatives promoting active transportation, such as the German National Cycling Plan, they may instead reflect broader societal developments. Rising overall mobility demands may be contributing simultaneously to increases in both active transportation and transport-related sitting time. Future research should explore physical behavior within the transportation context more closely to better understand these underlying patterns and their implications.

However, it should be noted that adherence to the WHO physical activity guidelines in our surveys (80–88%) is markedly higher than national estimates for Germany [7]. Direct comparison, however, is limited because national surveillance systems use instruments that differ considerably from GPAQ: they generally exclude occupational physical activity, capture transport activity only partially, and use broader leisure-time-focused questions [42]. Each of these methodological features tends to yield lower estimates of guideline adherence. Consistent with the higher adherence observed in our surveys, GPAQ-based results from the German National Cohort report comparatively high MVPA volumes (mean 194–244 min per day across age groups), although the initial German National Cohort report did not provide guideline-adherence prevalence [43]. GPAQ-based data from other European populations show similarly elevated physical activity levels [44]. Taken together, the high prevalence of adherence in our surveys likely reflects methodological differences in physical activity assessment rather than the inclusion of a genuinely more active population. Nevertheless, if our participants are more active than the general population, the increase in sedentary time observed in our study may underestimate the true secular trend in the broader German population.

Yet, it is important to note that high adherence to physical activity guidelines does not preclude prolonged sitting [45]. Individuals may meet recommended levels of moderate-to-vigorous physical activity while still spending a large portion of the day sedentary. Thus, the observed increase in sitting time can occur even in populations with high physical activity, highlighting the need to address sedentary behavior as an independent target for public health interventions in addition to promoting physical activity.

### Correlates of overall and domain-specific sitting time

Sitting time is associated with various sociodemographic factors, which vary across different contexts of sitting, as also reflected in our findings. These results suggest that public health action is needed to reduce sitting time, considering both the target population and the context in which prolonged sitting occurs.

The evidence regarding gender differences in sitting time remains inconclusive. Several studies report that men tend to sit longer in the context of transport [16, 46], a finding that is consistent with our results. However, when considering overall sitting time and leisure sitting, a systematic review suggests that women tend to sit longer [16]. Our findings support this for leisure time, as women reported sitting approximately six minutes longer per weekday during leisure activities than men. In contrast, studies from Europe, particularly Germany, indicate that men have higher overall sitting times [9, 29, 31, 47], a pattern also reflected in our results. A possible explanation is that women in Germany are more likely to work part-time and bear the primary responsibility for family duties [48]. This may result in lower occupational sitting time - one of the main contributors to overall sitting - while potentially increasing leisure time sitting. Broader labor market shifts across Europe — including the decline of physically demanding occupations and the rise of service- and technology-based work — may also have contributed to higher sedentary time, particularly among men, who may be more affected by these structural changes [38].

Overall, age was inversely associated with overall sitting time, contrasting with findings from a previous systematic review [16]. This negative association in our results is particularly pronounced in occupational sitting time, which appears plausible given that older individuals are more likely to work fewer hours or retire. Consistent with previous research, our findings indicate that sitting time related to TV viewing [46, 49] and leisure activities [46] increases with age. These differences may also, at least in part, be explained by our domain-specific measurement approach, whereas other studies often rely on single-item assessments of overall sitting time. Thus, discrepancies in age-related patterns may also partly reflect differences in assessment methods. Future research with the existing data may also benefit from specifically examining sitting patterns in individuals aged 65 and older as a proxy for retirement. This could help determine whether older adults exhibit similar sitting time trends as reported in the existing literature. To enhance the validity of future data collection in older adults, the use of self-report questionnaires specifically designed for this population could also be beneficial [50].

The primary driver of the positive association of BMI with overall sitting time in our results appears to be TV-related sitting time. Increasing TV-related sitting time with a higher BMI, lower educational and income level is a well-documented phenomenon [16] and is reflected in our findings. However, we mainly observe a positive association between educational and income level and overall sitting time, particularly in the work-related context, which aligns with the sedentary nature of desk-based occupations that are more common among individuals with higher education and income. This finding is consistent with previous research [15–17, 51] and highlights the urgent need for targeted workplace interventions to reduce sitting time in desk-based settings.

Among environmental correlates, it has been shown in the European context that those living in urban areas are more likely to have high sitting times [9, 17], which is also true for our results looking at overall, electronic-media-related, and leisure-related sitting time. This may be partly explained by the higher prevalence of sedentary office-based occupations in cities, with many of those employed in such roles also residing in the urban environment—though it has been acknowledged that also a significant number commute from outside the city. Furthermore, higher leisure sitting times may be explained through limited access to private green spaces, which may encourage more indoor, screen-based leisure activities. Interestingly, no clear pattern emerged for transport-related sitting time in relation to urbanity. Participants from mid-sized towns reported a three-minute lower mean transport-related sitting time compared to those from towns with fewer than 20,000 inhabitants, while no significant association was observed in urban areas. This may be explained by factors such as the high volume of traffic and safety concerns that may discourage active mobility and promote passive transportation. However, cities most often have a better infrastructure for active mobility. Yet, this phenomenon warrants further investigation.

Regarding physical activity, an inverse association with sitting time has been reported [9, 17, 52], which is also reflected in our findings for overall, work-related, and TV-related sitting time.

### Strength and limitations

Strengths of this study include the use of a serial, nationally representative survey spanning nine years among German adults with the same methodological assessment, which also allows observation of trends before, during, and after the COVID-19 pandemic. However, some limitations should be acknowledged. Estimates of sitting time and physical activity rely on self-reported data, which may be affected by measurement error [30] due to recall bias, social desirability bias, or misclassification, potentially leading participants to underestimate sitting time and overestimate physical activity. Nevertheless, we utilized a multi-item questionnaire, which has greater validity compared to the commonly used single-item measures [30] but makes comparison between other studies difficult. However, we only assessed sitting time for weekdays, which may not capture different patterns of sitting time during weekends. Furthermore, while the survey aims to provide representative data, variations in the sample composition across years were observed, particularly within socio-demographic groups (e.g., education), introducing a small selection bias, potentially leading to over- or underestimation of sitting time depending on the domain. However, these differences were accounted for through weight adjustments. Third, the low response rates in the series of surveys are a further limitation. This could have potentially been caused by the overall mean length of more than 20 min of the surveys covering a broad range of health topics, which could have resulted in a high non-response rate. Considering the methodology-related literature on surveys [53], the response rates in this study remain acceptable for addressing the stated research question.

## Conclusion

Since 2014, overall weekday sitting time has steadily and substantially increased among German adults, primarily driven by work-related, transport-related, and leisure electronic media use. These findings highlight the urgent need for targeted, evidence-based interventions that reduce sitting time across the key domains of work, transport, and leisure. Health policies should prioritize the development and implementation of context-specific strategies, including workplace redesign to encourage standing and movement, promotion of active commuting, and initiatives to limit screen time during leisure. Additionally, tailored approaches are necessary to address the needs of specific population groups identified as particularly at risk.

## Supplementary Information

Supplementary Material 1.

## Data Availability

The datasets used and analyzed during the current study are available from the corresponding author on reasonable request.

## Abbreviations

METs: Metabolic equivalent
MVPA: Moderate–to–vigorous physical activity
CATI: Computer–assisted telephone interview
ADM: Arbeitskreis Deutscher Markt–und Sozialforschungsinstitute
GPAQ: Global Physical Activity Questionnaire
IPAQ: International Physical Activity Questionnaire
WHO: World Health Organization
BMI: Body mass index
MICE: Multivariate Imputation by Chained Equations
