# Supplementary material for: Temporal trends and correlates of overall and domain-specific sitting time in Germany between 2014 and 2023
Source: BMC Public Health. 2025 Dec 30;26:730. doi: 10.1186/s12889-025-26005-9 (PMC12930690; doi:10.1186/s12889-025-26005-9)
Supplement: Supplementary file 1 — Supplementary Material 1. [file 12889_2025_26005_MOESM1_ESM.docx]

**Supplement**

**Table S1.** Missing values by survey year.

| Year | Income | Overall sitting | Transport | Work | Television | Electronic media use at home | Leisure time, other |
| --- | --- | --- | --- | --- | --- | --- | --- |
| 2014 | 410 (13%) | 181 (6%) | 183 (6%) | 181 (6%) | 183 (6%) | 181 (6%) | 184 (6%) |
| 2016 | 1676 (59%) | 138 (5%) | 138 (5%) | 139 (5%) | 139 (5%) | 139 (5%) | 139 (5%) |
| 2018 | 1399 (48%) | 133 (5%) | 134 (5%) | 133 (5%) | 134 (5%) | 134 (5%) | 134 (5%) |
| 2021 | 1218 (43%) | 303 (11%) | 303 (11%) | 303 (11%) | 303 (11%) | 303 (11%) | 303 (11%) |
| 2023 | 1456 (52%) | 17 (< 1%) | 17 (< 1%) | 17 (< 1%) | 17 (< 1%) | 17 (< 1%) | 17 (< 1%) |

**Table S2:** Unweighted sample demographic characteristics among the serial cross-sectional studies from 2014 to 2023.

| **Characteristic** | **2014** N = 3,102*^1^* | **2016** N = 2,830*^1^* | **2018** N = 2,885*^1^* | **2021** N = 2,800*^1^* | **2023** N = 2,800*^1^* |
| --- | --- | --- | --- | --- | --- |
| Sex |  |  |  |  |  |
| Female | 1,668 (54%) | 1,636 (58%) | 1,725 (60%) | 1,571 (56%) | 1,557 (56%) |
| Male | 1,434 (46%) | 1,194 (42%) | 1,160 (40%) | 1,229 (44%) | 1,243 (44%) |
| Age |  |  |  |  |  |
| 18-29 | 268 (8.6%) | 236 (8.3%) | 227 (7.9%) | 264 (9.4%) | 138 (4.9%) |
| 30-44 | 761 (25%) | 712 (25%) | 432 (15%) | 556 (20%) | 402 (14%) |
| 45-64 | 1,322 (43%) | 1,097 (39%) | 1,272 (44%) | 1,170 (42%) | 1,190 (43%) |
| 65-79 | 627 (20%) | 626 (22%) | 757 (26%) | 635 (23%) | 770 (28%) |
| 80+ | 124 (4.0%) | 159 (5.6%) | 197 (6.8%) | 175 (6.3%) | 300 (11%) |
| Body Mass Index (WHO categories) |  |  |  |  |  |
| Underweight | 51 (1.7%) | 125 (4.5%) | 65 (2.3%) | 68 (2.4%) | 55 (2.0%) |
| Normal weight | 1,458 (48%) | 1,328 (47%) | 1,360 (49%) | 1,393 (50%) | 1,392 (50%) |
| Overweight | 454 (15%) | 364 (13%) | 422 (15%) | 419 (15%) | 408 (15%) |
| Obesity | 1,081 (36%) | 990 (35%) | 951 (34%) | 912 (33%) | 938 (34%) |
| Unknown | 58 | 23 | 87 | 8 | 7 |
| Current Smoking | 663 (21%) | 576 (20%) | 528 (18%) | 585 (21%) | 420 (15%) |
| Physical activity guideline adherence | 2,654 (88%) | 2,253 (81%) | 2,307 (80%) | 2,278 (81%) | 2,344 (84%) |
| Unknown | 75 | 61 | 14 | 1 | 26 |
| Education |  |  |  |  |  |
| No educational qualification | 22 (0.7%) | 38 (1.3%) | 33 (1.1%) | 43 (1.5%) | 23 (0.8%) |
| Lower secondary school (9 yrs) | 506 (16%) | 378 (13%) | 317 (11%) | 324 (12%) | 257 (9.2%) |
| Secondary school (10 yrs) | 1,116 (36%) | 932 (33%) | 961 (33%) | 671 (24%) | 701 (25%) |
| High school or university (≥13 yrs) | 1,458 (47%) | 1,482 (52%) | 1,574 (55%) | 1,762 (63%) | 1,819 (65%) |
| Household income (€ per month) |  |  |  |  |  |
| <2,000 | 1,427 (53%) | 404 (35%) | 564 (38%) | 532 (34%) | 264 (20%) |
| 2,000-<4,000 | 959 (36%) | 545 (47%) | 654 (44%) | 677 (43%) | 640 (48%) |
| ≥4,000 | 306 (11%) | 205 (18%) | 268 (18%) | 373 (24%) | 440 (33%) |
| Unknown | 410 | 1,676 | 1,399 | 1,218 | 1,456 |
| Community size (no. of inhabitants) |  |  |  |  |  |
| <20,000 | 1,345 (43%) | 1,172 (41%) | 1,169 (41%) | 1,143 (41%) | 1,221 (44%) |
| 20,000-500,000 | 1,145 (37%) | 1,061 (37%) | 1,097 (38%) | 965 (34%) | 1,013 (36%) |
| >500,000 | 612 (20%) | 597 (21%) | 619 (21%) | 692 (25%) | 566 (20%) |
| *^1^*n (%) | | | | | |

**Table S3.** Overall and domain-specific sitting time and prevalence of high sitting time (>8 hours/day) among adults in Germany across survey years 2014 to 2023 (minutes per day, 95% confidence intervals) for the unweighted sample.

| **Domain** | **2014** N = 3,102 (**95% CI**) | **2016** N = 2,830 (**95% CI**) | **2018** N = 2,885 (**95% CI**) | **2021** N = 2,800 (**95% CI**) | **2023** N = 2,800 (**95% CI**) |
| --- | --- | --- | --- | --- | --- |
| Overall | 465  (458, 473) | 457  (449, 465) | 466  (458, 473) | 525  (516, 533) | 524  (516, 532) |
| Transport | 47  (45, 49) | 43  (41, 45) | 46  (44, 48) | 47  (44, 49) | 52  (49, 54) |
| Work | 136  (131, 142) | 126  (119, 132) | 140  (134, 147) | 167  (160, 174) | 172  (166, 179) |
| Television | 120  (117, 123) | 120  (116, 123) | 117  (114, 120) | 123  (120, 126) | 119  (116, 122) |
| Electronic media use at home | 59  (56, 62) | 61  (58, 63) | 60  (57, 63) | 82  (79, 85) | 76  (73, 78) |
| Leisure time, other | 103  (100, 106) | 109  (105, 112) | 102  (99, 105) | 106  (103, 109) | 105  (103, 108) |
| Prevalence >8h sitting  (%) | 43%  (41%, 45%) | 41%  (39%, 43%) | 43%  (41%, 45%) | 54%  (52%, 56%) | 53%  (51%, 55%) |

**Table S4**. Comparison of fully adjusted regression models: linear effect vs. natural cubic splines for survey year.

| **Domain** | **AIC** | | **Likelihood-ratio-test** |
| --- | --- | --- | --- |
|  | **Linear term** | **Spline term** |  |
| Overall sitting | 102,775 | 102,774 | 0.101 |
| Transport | 84,503 | 84,509 | 0.738 |
| Work | 98,465 | 98,469 | 0.321 |
| Television | 86,707 | 86,698 | **0.011** |
| Electronic media use at home | 86,790 | 86,738 | **< 0.001** |
| Leisure time, other | 87,254 | 87,250 | 0.052 |

**Table S5**. McFadden’s pseudo R² for each regression model.

| **Overall sitting** | **Transport** | **Work** | **Television** | **Electronic media  use at home** | **Leisure time, other** |
| --- | --- | --- | --- | --- | --- |
| 0.12 | 0.03 | 0.24 | 0.15 | 0.09 | 0.02 |

**Table S6**. Generalised Variance Inflation Factors (GVIF) for each predictor variable and model.

|  | **Overall sitting** | **Transport** | **Work** | **Television** | **Electronic media  use at home** | **Leisure time, other** |
| --- | --- | --- | --- | --- | --- | --- |
|  | GVIF (mean [min - max]) | | | | | |
| **Year** | 1.08 [1.08 - 1.09] | 1.18 [1.17 - 1.2] | 1.13 [1.13 - 1.14] | 1.28 [1.26 - 1.3] | 1.31 [1.29 - 1.35] | 1.09 [1.08 - 1.11] |
| **Sex** | 1.06 [1.05 - 1.07] | 1.11 [1.09 - 1.13] | 1.06 [1.05 - 1.07] | 1.11 [1.1 - 1.13] | 1.12 [1.09 - 1.14] | 1.14 [1.13 - 1.15] |
| **Age** | 1.27 [1.27 - 1.28] | 1.28 [1.26 - 1.3] | 1.42 [1.4 - 1.44] | 1.3 [1.29 - 1.32] | 1.5 [1.46 - 1.54] | 1.08 [1.07 - 1.09] |
| **BMI** | 1.07 [1.07 - 1.08] | 1.12 [1.12 - 1.13] | 1.09 [1.08 - 1.09] | 1.09 [1.08 - 1.1] | 1.27 [1.23 - 1.3] | 1.14 [1.14 - 1.14] |
| **Education** | 1.51 [1.49 - 1.54] | 1.56 [1.5 - 1.62] | 1.64 [1.58 - 1.66] | 1.53 [1.49 - 1.63] | 1.82 [1.69 - 2.17] | 1.33 [1.3 - 1.38] |
| **Income** | 1.27 [1.25 - 1.28] | 1.35 [1.3 - 1.38] | 1.34 [1.32 - 1.36] | 1.27 [1.21 - 1.31] | 1.4 [1.31 - 1.6] | 1.19 [1.15 - 1.27] |
| **Inhabitants** | 1.06 [1.05 - 1.06] | 1.05 [1.04 - 1.06] | 1.05 [1.05 - 1.06] | 1.07 [1.07 - 1.08] | 1.13 [1.12 - 1.14] | 1.08 [1.07 - 1.08] |
| **MVPA (minutes per day)** | 1.07 [1.07 - 1.07] | 1.04 [1.04 - 1.07] | 1.13 [1.13 - 1.14] | 1.13 [1.13 - 1.14] | 1.21 [1.2 - 1.22] | 1.06 [1.06 - 1.08] |

**Table S7.** Regression coefficients using only complete cases.

|  | **Overall**  **sitting** | **Transport** | **Work** | **Television** | **Electronic media use at home** | **Leisure time, other** |
| --- | --- | --- | --- | --- | --- | --- |
| **Calendar year** | 10.27  (8.36 , 12.18) | 0.8  (0.26 , 1.34) | 8.55  (7.18 , 9.92) | -0.81 (-1.37 , -0.24) | 0.64 (0.08 , 1.21) | -0.53  (-1.19 , 0.14) |
| **Age (years)** | -1.37  (-1.82 , -0.92) | -0.03  (-0.16 , 0.11) | -1.7  (-2.02 , -1.39) | 1.07 (0.89 , 1.25) | -0.87 (-1.04 , -0.7) | 0.17  (-0.01 , 0.34) |
| **Male** | 35.59  (23.37 , 47.81) | 13.68  (10.18 , 17.19) | 11.87  (3.01 , 20.72) | 1.35 (-3.07 , 5.76) | 14.75 (10.49 , 19.01) | -5.91  (-10.49 , -1.33) |
| **Body mass index  (kg/m2)** | 2.65  (1.56 , 3.74) | -0.16  (-0.5 , 0.17) | 0.34  (-0.42 , 1.11) | 1.98 (1.51 , 2.44) | 0.74 (-0.01 , 1.49) | -0.2  (-0.68 , 0.28) |
| **Secondary school  (10 years)** | 10.11  (-9.05 , 29.26) | 0.58  (-5.06 , 6.22) | 24.48  (11.07 , 37.88) | -10.5 (-17.57 , -3.44) | 0.21 (-6.06 , 6.48) | -3.68  (-10.62 , 3.26) |
| **High school or university  (≥13 years)** | 50.72  (31.41 , 70.02) | -2.43  (-7.93 , 3.07) | 70.11  (56.32 , 83.89) | -25.02 (-32.08 , -17.96) | 15.31 (8.76 , 21.86) | -6.62  (-13.3 , 0.07) |
| **No formal educational qualification** | -64.44  (-126.05 , -2.84) | -22.37  (-32.25 , -12.48) | -44.71  (-77.92 , -11.5) | -8.66 (-34.99 , 17.67) | 11.87 (-9.43 , 33.17) | -0.87  (-27.63 , 25.89) |
| **Mid-sized town  (20.000-500,000 inhabitants)** | 10.14  (-3.39 , 23.66) | -2.69  (-6.56 , 1.18) | 4.62  (-5.21 , 14.45) | 1.21 (-3.68 , 6.11) | 4.34 (-0.21 , 8.89) | 2.4  (-2.64 , 7.44) |
| **Large city**  **(>500,000 inhabitants)** | 29.02  (12.08 , 45.96) | -0.23  (-5.5 , 5.05) | 13.07  (1.32 , 24.81) | -1.04 (-6.92 , 4.85) | 8.54 (2.66 , 14.42) | 8.43  (2.33 , 14.54) |
| **2,000-<4,000€ monthly income** | 11.89  (-1.92 , 25.7) | 3.14  (-0.99 , 7.26) | 34.01  (24.05 , 43.97) | -11.76 (-16.62 , -6.89) | -5.68 (-10.22 , -1.13) | -5.86  (-10.75 , -0.97) |
| **≥4,000€ monthly income** | 40.97  (23.62 , 58.31) | 5.11  (-0.16 , 10.39) | 80.02  (66.26 , 93.77) | -22.38 (-27.94 , -16.82) | -6.85 (-13.23 , -0.47) | -13.08  (-18.73 , -7.42) |
| **MVPA (minutes per day)** | -0.21 (-0.24 , -0.17) | 0.02 (0.01 , 0.03) | -0.21 (-0.23 , -0.19) | -0.01 (-0.02 , 0) | -0.01 (-0.02 , 0) | 0 (-0.01 , 0.02) |
